# DDA-SKF: Predicting Drug–Disease Associations Using Similarity Kernel Fusion

**DOI:** 10.3389/fphar.2021.784171

**Published:** 2022-01-13

**Authors:** Chu-Qiao Gao, Yuan-Ke Zhou, Xiao-Hong Xin, Hui Min, Pu-Feng Du

**Affiliations:** College of Intelligence and Computing, Tianjin University, Tianjin, China

**Keywords:** drug repositioning, drug–disease association, similarity kernel fusion, Laplacian regularized least squares, orphan drugs

## Abstract

Drug repositioning provides a promising and efficient strategy to discover potential associations between drugs and diseases. Many systematic computational drug-repositioning methods have been introduced, which are based on various similarities of drugs and diseases. In this work, we proposed a new computational model, DDA-SKF (drug–disease associations prediction using similarity kernels fusion), which can predict novel drug indications by utilizing similarity kernel fusion (SKF) and Laplacian regularized least squares (LapRLS) algorithms. DDA-SKF integrated multiple similarities of drugs and diseases. The prediction performances of DDA-SKF are better, or at least comparable, to all state-of-the-art methods. The DDA-SKF can work without sufficient similarity information between drug indications. This allows us to predict new purpose for orphan drugs. The source code and benchmarking datasets are deposited in a GitHub repository (https://github.com/GCQ2119216031/DDA-SKF).

## 1 Introduction

In recent decades, although investment in pharmaceutical research and development has increased substantially, the discovery of a new drug is still a time-consuming, risky, and challenging process (Chong and Sullivan, 2007; Paul et al., 2010; Pammolli et al., 2011; Li et al., 2016). The pharmaceutical industry did not receive rational benefits from investments (Kola and Landis, 2004; Munos, 2009; Schuhmacher et al., 2016). A large number of new drug candidates failed in the FDA evaluations, thereby preventing their applications in therapies (Weng et al., 2013; Mullard, 2017; Mullard, 2018; Mullard, 2019; Mullard, 2020; Mullard, 2021). Today, drug repositioning has gained importance in identifying new therapeutic purposes for already-approved drugs (Parvathaneni et al., 2019). Repositioning of “old” drugs to treat both common and rare diseases is becoming an attractive proposition because it involves the use of safe compounds, with potentially lower overall development costs and shorter development cycles (Pushpakom et al., 2019). So far, drug repositioning has achieved many successes (Swanson, 1990; Soignet et al., 1998; Ashburn and Thor, 2004). Currently, rare diseases are a serious threat to human health (Wästfelt et al., 2006; Harari, 2016). Several orphan drugs are intended to treat rare diseases. Developing a new drug intended to treat a rare disease is costly and time-consuming (Attwood et al., 2018). Drug repositioning is considered as a more efficient strategy than traditional drug development (Scherman and Fetro, 2020). In fact, some successfully repurposed orphan drugs (Gordon et al., 2018; Kurolap et al., 2019; Scherman and Fetro, 2020) have attracted much attention. Therefore, systematic computational drug repositioning is an important research topic in both medical science and computational life science.

Many computational methods have been proposed to predict potential drug–disease associations for drug repositioning. Machine learning approaches play important roles in predicting associations between drugs and diseases. For example, Gottlieb et al. (2011) integrated several drug similarities and disease similarities to score the novel drug–disease associations by learning a logistic regression classifier. Recently, a method, which is called DRIMC (Zhang et al., 2020), introduced a drug-repositioning approach by using Bayesian inductive matrix completion. Xuan et al. (2019) presented DisDrugPred method based on nonnegative matrix factorization to predict the drug-related candidate indications. SCMFDD (Zhang et al., 2018b) proposed a similarity-constrained matrix factorization method for the drug–disease association prediction. PREDICT (Gottlieb et al., 2011) integrated several similarities to score the novel associations by learning a logistic regression classifier. LRSSL (Liang et al., 2017) proposed a Laplacian regularized sparse subspace learning method to identify novel indications.

Recently, the systems biology–based methods have been overwhelmingly studied in predicting drug–disease associations. Most methods of this kind constructed a heterogenous network model before calculating prediction scores. The MBiRW model (Luo et al., 2016) utilized bi-random walk (BiRW) algorithm to predict unknown drug–disease associations on a heterogenous network. Luo et al. (2018) designed a drug repositioning recommendation system (DRRS) based on the singular value decomposition to complete the potential association matrix of the heterogenous network. BNNR (Yang et al., 2019a) employs a bounded norm regularization method to complete the drug–disease matrix under the low-rank assumption. NTSIM (Zhang et al., 2018a) used the network topological similarity-based inference method. Yu et al. (2021) proposed a novel computational method, which is named as a layer attention graph convolutional network, to predict unobserved associations.

Previous studies have achieved many successes. It should be noted that most existing methods employ only one type of drug or disease similarity rather than integrating multiple similarities (Yang et al., 2019a; Yang et al., 2019b). Some models utilized linear combinations to integrate multiple similarities (Jiang et al., 2019; Yan et al., 2019), which loses the high-order interactions between different similarities.

**FIGURE 1:**
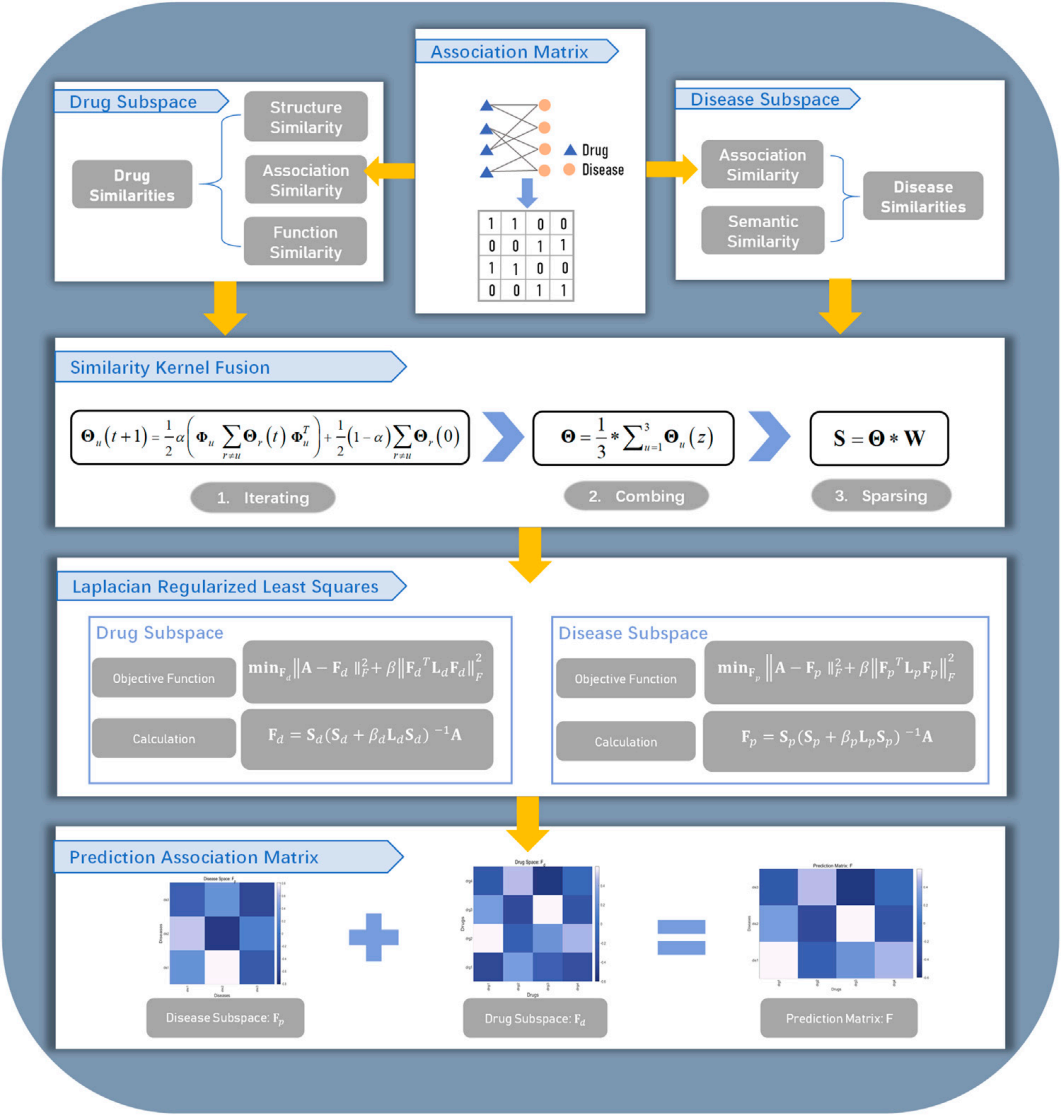
Flowchart of DDA-SKF. The model DDA-SKF included three steps: (1) three drug similarity kernels and two disease similarity kernels were calculated; (2) similarity kernel fusion (SKF) algorithm was used to integrate these similarities into two comprehensive similarity kernels; (3) Laplacian regularized least squares (LapRLS) framework was used to build the prediction model.

In this work, we proposed the similarity kernel fusion (SKF) to integrate different similarity kernels with Laplacian regularized least squares (LapRLS) algorithms. We named our method as the DDA-SKF method. The overall workflow of DDA-SKF was illustrated in Figure 1. First, we constructed multiple similarity kernels for drugs and diseases. These similarity kernels were integrated by the SKF iterative process into two comprehensive similarity kernels. Finally, the Laplacian regularized least squares (LapRLS) algorithms were used to obtain the prediction association matrix. To demonstrate the effectiveness of our model, we compared it with several state-of-the-art methods.

## 2 Materials and Methods

### 2.1 Drug–Disease Association Dataset

We adapted two benchmarking datasets in this article, which are the PREDICT dataset and the LRSSL dataset. The PREDICT dataset was obtained from the literature (Gottlieb et al., 2011), which was originally collected from the DrugBank database (Wishart et al., 2018) and the OMIM (Online Mendelian Inheritance in Man) database (Hamosh et al., 2005). It contains 1933 known drug–disease associations between 593 drugs and 313 diseases. The LRSSL dataset was extracted from the literature (Liang et al., 2017). It contains 3,051 drug–disease associations between 763 drugs and 681 diseases.

Without losing generality, we take the PREDICT dataset as an example to describe our method. Let *p*_*i*_ (*i* = 1, 2, … , 313) be the *i*-th disease and *d*_*j*_ (*j* = 1, 2, … , 593) the *j*-th drug. We defined the relationship between *p*_*i*_ and *d*_*j*_ as:

$$a_{ij}=\{\begin{array}{cc} 0 & p_{i}\text{ and }d_{j}\text{ have no association,}\\ 1 & otherwise \end{array}.$$ (1)

An adjacency matrix was then created to describe the drug–disease associations in the whole dataset which can be noted as **A** ∈ **R** ^313×593^.

### 2.2 Similarity Kernels for Drug and Disease

The basic assumption of this work is that drug-related diseases are more likely to be similar when the drugs are more similar. Three different similarity kernels of drugs and two different similarity kernels of diseases were applied. By fusing these similarity kernels, our method can identify potential associations between drugs and diseases.

#### 2.2.1 Drug Chemical Similarity

We obtained the drug chemical similarity kernel from the literature (Zhang et al., 2020). PaDEL software (Yap, 2011) was used to compute PubChem fingerprint descriptors for each drug. The pairwise similarity between drugs was measured by the Jaccard coefficient between PubChem fingerprints. The drug chemical similarity kernel was noted as a 593 × 593 matrix **M**_*d*,1_. **M**_*d*,1_ (*i*, *j*), which is the element in the *i*-th row and the *j*-th column of **M**_*d*,1_, represents the chemical similarity between the *i*-th and the *j*-th drug. Because of the Jaccard coefficient definition, 0 ≤ **M**_*d*,1_ (*i*, *j*) ≤ 1.

#### 2.2.2 Drug Functional Similarity Kernel

The drug function was described by the Gene Ontology terms of the target genes. The Jaccard coefficient was used to measure the similarity between the Gene Ontology annotations of two different target genes. We obtain the similarity values from the literature (Zhang et al., 2020). The drug functional similarity was noted also as a 593 × 593 matrix **M**_*d*,2_. The element **M**_*d*,2_ (*i, j*) is the functional similarity between the *i*-th drug and the *j*-th drug.

#### 2.2.3 Association Similarity Kernels for Drugs and Diseases

The association profile of the *i-*th drug is defined as the *i-*th column of the association matrix **A**, which can be noted as A_*i_. The drug association similarity kernel was represented by a 593 × 593 matrix **M**_*d*,3_; the element **M**_*d*,3_ (*i, j*) is the association similarity between *d*_*i*_ and *d*_*j*_, which can be defined as:

$$M_{d,3}\left(i,j\right)=\exp\left(-\gamma_{d}{\left\|A_{\text{*}i}-A_{*j}\right\|}^{2}\right),$$ (2)

where

$$\gamma_{d}=593/\sum_{i=1}^{593}{\left\|A_{*i}\right\|}^{2},$$ (3)

and ||.|| is the 2-norm operator.

Similarly, the association profile of the *i-*th disease is defined as the *i-*th row of the association matrix **A**, which can be noted as **A**_i*_. The disease association similarity kernel was represented by a 313 × 313 matrix **M**_*p*,1_; the element **M**_*p*,1_ (*i, j*) is the association similarity between disease *p*_*i*_ and disease *p*_*j*_, which can be calculated as:

$$M_{p,1}\left(i,j\right)=\exp\left(-\gamma_{p}{\left\|A_{i*}-A_{j*}\right\|}^{2}\right),$$ (4)

where

$$\gamma_{p}=313/\sum_{i=1}^{313}{\left\|A_{i*}\right\|}^{2},$$ (5)

#### 2.2.4 Disease Semantic Similarity Kernel

A disease semantic similarity kernel is a 313 × 313 matrix **M**_*p*,2_. The element **M**_*p*,2_ (*i*, *j*) is the similarity between disease *p*_*i*_ and disease *p*_*j*_. The disease semantic similarity is accessible by MimMiner (van Driel et al., 2006), which is a text mining approach to quantify phenotype relationships between human disease phenotypes from the OMIM database. We obtain the values of **M**_*p*,2_ from the literature (Zhang et al., 2020).

### 2.3 Similarity Kernel Fusion

The similarity kernel fusion (SKF) algorithm was applied to integrate three drug similarity kernels into a drug comprehensive similarity kernel and two disease similarity kernels into a disease comprehensive similarity kernel. Without losing generality, we take the drug similarity kernels as an example to explain this fusing process.

First, we normalize the aforementioned three drug similarity kernels (**M**_*d*,*u*_, *u* = 1, 2, 3) by using the following formula:

$$\theta_{d,u}\left(i,j\right)=\frac{M_{d,u}\left(i,j\right)}{\sum_{v=1}^{593}M_{d,u}\left(v,j\right)},$$ (6)

where *θ*_*d,u*_ (*i*, *j*) represents a normalized similarity corresponding to **M**_*d*,*u*_ (*i*, *j*). The matrix composed by the normalized kernel is noted as:

$$\Theta_{d,u}={\left\{\theta_{d,u}\left(i,j\right)\right\}}_{593\times 593},$$ (7)

Second, we established a neighbor-constrained normalization kernel for each drug similarity kernel. Given the drug *d*_*i*_ and **M**_*d*,*u*_, we collected the *k* most similar drugs as a set *N*_*d,u*_ (*i, k*). The neighborhood-constrained normalization of the **M**_*d*,*u*_ can be defined as follows:

$$\varphi_{d,u}\left(i,j\right)=\frac{M_{d,u}\left(i,j\right)I_{d,u,k}\left(i,j\right)}{\sum_{v=1}^{593}M_{d,u}\left(i,v\right)I_{d,u,k}\left(i,v\right)},$$ (8)

where

$$I_{d,u,k}\left(i,j\right)=\left\{ \begin{array}{l} 1\;d_{j}\in N_{d,u}\left(i,k\right)\\ 0\;d_{j}\notin N_{d,u}\left(i,k\right) \end{array}\right.,$$ (9)

The matrix composed by the neighborhood-constrained normalization is noted as:

$$\Phi_{d,u}={\left\{\varphi_{d,u}\left(i,j\right)\right\}}_{593\times 593},$$ (10)

Finally, the normalized kernel **Θ**_*d,u*_ and the neighbor-constrained normalization kernel **Φ**_*d,u*_ were fused by using the following iterative process:

$$\Theta_{d,u}\left(t+1\right)=\frac{1}{2}\alpha \left(\Phi_{d,u}\;\sum_{r\neq u}\Theta_{d,r}\left(t\right)\;\Phi_{d,u}^{T}\right)+\frac{1}{2}\left(1-\alpha \right)\sum_{r\neq u}\Theta_{d,r}\left(0\right),$$ (11)

where *t* is the number of iterations, *α*, a weight parameter between 0 and 1, *T*, the transpose operator in matrix algebra, and

$$\Theta_{d,r}\left(0\right)=\Theta_{d,r}.$$ (12)

After *z* rounds of iterative computations, we obtained the integration kernel as follows:

$$\Theta_{d}=\frac{1}{3}\left(\Theta_{d,1}\left(z\right)+\Theta_{d,2}\left(z\right)+\Theta_{d,3}\left(z\right)\right),$$ (13)

Although useful information was extracted in the fusion process, noise is inevitable simultaneously. The information of *k* most similar drugs for each drug was further extracted to suppress the noise influence by defining a weight matrix; we defined an indicator function as follows:

$$w_{d,k}\left(i,j\right)=\left\{ \begin{array}{l} \;1\;I_{d,1,k}\left(i,j\right)=I_{d,2,k}\left(i,j\right)=I_{d,3,k}\left(i,j\right)=1\\ \;0\;I_{d,1,k}\left(i,j\right)=I_{d,2,k}\left(i,j\right)=I_{d,3,k}\left(i,j\right)=0\\ 0.5\;otherwise \end{array}\right.,$$ (14)

Finally, the drug comprehensive similarity kernel was defined as the following formula:

$$S_{d,k}={\left\{\theta_{d}\left(i,j\right)w_{d,k}\left(i,j\right)\right\}}_{593\times 593},$$ (15)

where *θ*_*d*_ (*i*, *j*) is the element in the *i*-th row and the *j*-th column of the matrix **Θ**_*d*_.

By applying Eqs 6–15 and employing two disease similarities, we obtained the disease comprehensive similarity kernel **S**_*p,k*_. The value of *k* in the computing disease comprehensive similarity kernel is not necessarily the same as that of the drugs.

### 2.4 Laplacian Regularized Least Squares

In this work, Laplacian regularized least squares (LapRLS) were employed to build the prediction model to uncover the potential drug–disease associations. Based on the characteristics of the model, we could predict the drug–disease associations from either the drug subspace or disease subspace.

In terms of the drug subspace, the Laplacian similarity matrix **L**_*d*_ was represented as follows:

$$L_{d}=D_{d}^{-1/2}\left(D_{d}-S_{d,k}\right)D_{d}^{-1/2},$$ (16)

where **D**_*d*_ is a diagonal matrix whose diagonal elements are the sum of the corresponding row elements of the matrix **S**_*d,k*_.

The drug–disease association prediction matrix **F**_*d*_ was calculated by minimizing the following loss function:

$$\underset{F_{d}}{\min}\left({\left\|A-F_{d}\right\|}_{F}^{2}+\beta_{d}{\left\|F_{d}^{T}L_{d}F_{d}\right\|}_{F}^{2}\right),$$ (17)

where **A** is the original drug–disease association matrix, **F**_*d*_, the predicted association matrix in the drug subspace, *β*_*d*_, the weighting coefficient, and ||.||_*F*_, the F-norm operator. The first term of the loss function aims to reduce the difference between the original matrix and prediction matrix. The second term is used to avoid the over-fitting problem.

The derivation of the optimization algorithm of LapRLS was introduced in Xia et al. (2010). We can calculate the predicted association matrix in the drug subspace by using the following formula:

$$F_{d}=S_{d,k}{\left(S_{d,k}+\beta_{d}L_{d}S_{d,k}\right)}^{-1}A.$$ (18)

Similarly, by using Eqs 16–18 on the disease subspace, the predicted association matrix **F**_*p*_ in the disease subspace was calculated as follows:

$$F_{p}=S_{p,k}{\left(S_{p,k}+\beta_{p}L_{p}S_{p,k}\right)}^{-1}A,$$ (19)

where **L**_*p*_ is the normalized similarity matrix in the disease subspace. **L**_*p*_ was defined as follows:

$$L_{p}=D_{p}^{-1/2}\left(D_{p}-S_{p,k}\right)D_{p}^{-1/2},$$ (20)

where **D**_*p*_ is the diagonal matrix of the matrix **S**_*p,k*_.

With the two predicted association matrices **F**_*d*_ and **F**_*p*_, we defined the final predicted association matrix as follows:

$$F=\lambda F_{p}+\left(1-\lambda \right)F_{d},$$ (21)

where *λ* ∈ (0, 1) is a weighting parameter.

### 2.5 Performance Evaluation

We used novel association prediction and novel drug prediction to estimate the prediction performance of our method. In the novel association prediction, we applied both 5-fold and 10-fold cross-validation schemes. For the 10-fold cross validation, all drug–disease associations in the benchmarking dataset were divided into 10 nonoverlapping subsets randomly with almost the same size. While for the 5-fold cross validation, the number of the nonoverlapping subsets is five. The novel drug prediction was used to evaluate the prediction performance on new drugs. All drugs, not the drug–disease associations, were randomly divided into 10 subsets of approximately equal size. In each trial, one set was used in turn to act as the testing set, and other sets were used as the training set. In all aforementioned cross-validations, when one subset was used as the testing set, all prior knowledge of the testing set was removed before computing the association similarity kernels for drugs and diseases. The known associations corresponding to the testing set were reset to unknown. This guarantees that there is no information leak in the testing process.

All pairs of drug–disease associations were scored. Given a threshold, the drug–disease pair with a score larger than the threshold is predicted to be associated, otherwise nonassociated.

Due to the requirement of performance comparison and the performance values that are reported by the existing methods, we took a different set of performance measures in different contexts. We used the AUROC (area under receiver operating characteristics) curve and AUPR (area under precision-recall) curve to evaluate the prediction performance of our method in the context of 10-fold cross-validations. AUROC and AUPR are threshold-free metrics that are capable of measuring the overall performance of prediction models (Jiao and Du, 2016). While AUROC is powerful and popular as a main performance evaluation index, the value of AUROC may be misleading when the dataset is highly imbalanced (Saito and Rehmsmeier, 2015). It should be noticed that the value of AUPR can be used as an alternative metric to evaluate the prediction performance when the data is imbalanced (Davis and Goadrich, 2006). The value of AUPR tends to be smaller relative to the value of AUROC because of the highly imbalanced dataset (Saito and Rehmsmeier, 2015). In the context of 5-fold cross-validations, we applied several additional performance measures other than the AUROC and the AUPR. Six statistics, including sensitivity (SEN), specificity (SPE), precision (PRE), accuracy (ACC), F1-score (F1), and Matthew’s correlation coefficient (MCC), were applied in measuring the prediction performance of DDA-SKF in the context of 5-fold cross-validations. The threshold was optimized to maximize the F1-score. These performance measures were defined as follows:

$$SEN=\frac{TP}{TP+FN},$$ (22)

$$SPE=\frac{TN}{TN+FP},$$ (23)

$$PRE=\frac{TP}{TP+FP},$$ (24)

$$ACC=\frac{TN+TP}{TN+FN+TP+FP},$$ (25)

$$F1=\frac{2PRE\cdot SEN}{PRE+SEN},and$$ (26)

$$MCC=\frac{TP\cdot TN-FP\cdot FN}{\sqrt{\left(TP+FP\right)\left(TP+FN\right)\left(TN+FP\right)\left(TN+FN\right)}},$$ (27)

where *TP*, *TN*, *FP*, and *FN* represent the number of true positives, true negatives, false positives, and false negatives in the 5-fold cross-validation, respectively.

### 2.6 Parameter Calibration

There are three parameters in the SKF, which are the number of neighbors *k*, the weighting coefficient *α*, and the number of iterations *t*. There are also two parameters in the LapRLS, which are the weighting coefficients *β* and *λ*. To find the best parameter combination, we performed many trials manually with arbitrary values of parameters to optimize the AUROC value. We also performed a systematic exploration using a floating forward grid search strategy to further analyze the effects of different parameters. We finally fix the parameter values *α* and *k* as 0.2 and 15 in the drug subspace, while 0.7 and 10 in the disease subspace, respectively. The parameters *β* and *λ* were fixed as 2^−16^ and 0.4, respectively. The number of iteration *t* was determined using a stopping criterion. We defined the relative error *E*_*d*,*u*_ in the drug space as follows:

$$E_{d,u}\left(t\right)=\frac{∥\Theta_{d,u}\left(t+1\right)-\Theta_{d,u}\left(t\right)∥}{∥\Theta_{d,u}\left(t\right)∥},$$ (22a)

where *u* = 1, 2, 3. The relative error *E*_*p*,*u*_ in the disease subspace is defined similarly. In the disease subspace, if the *E*_*d*,*u*_ < 10^−7^ and *t* ≥ 10, the iteration will be terminated, while in the drug subspace, the iteration will be terminated if *E*_*p*,*u*_ <10^−10^ and *t* ≥ 10. The number of iterations was finally fixed as 10.

## 3 Results and Discussion

In this section, we verified the prediction performance of DDA-SKF. First, we compared the performance of DDA-SKF with other state-of-the-art methods. Second, case studies were conducted to confirm the effectiveness of DDA-SKF. Third, we employed single similarity in the drug and disease subspace to predict potential associations and compared the performance of single similarity and SKF.

### 3.1 Comparison With State-Of-The-Art Methods

In this section, to evaluate the prediction performance of our model on the benchmarking datasets using both novel association prediction and the novel drug prediction. We compared our model against other state-of-the-art methods, including BNNR (Yang et al., 2019a), DisDrugPred (Xuan et al., 2019), DRRS (Luo et al., 2018), SCMFDD (Zhang et al., 2018b), MBiRW (Luo et al., 2016), and DRIMC (Zhang et al., 2020). We also compared our method against the PREDICT (Gottlieb et al., 2011), LRSSL (Liang et al., 2017), and NTSIM methods (Zhang et al., 2018a).

To eliminate the randomness in the cross-validation, we repeated each cross-validation five times with different random data partition schemes. The average value and the standard deviations of every performance measures were reported. However, it should be noted that the standard deviations were not reported by other methods in comparison.

For the novel association prediction, the evaluation results of all the methods on the benchmarking dataset are collected in Table 1. As in Table 1, DDA-SKF obtained good values in AUROC and AUPR. DDA-SKF achieved an AUROC of 0.937, which is 0.536, 5.281, 0.861, 31.601, and 2.854%, respectively, higher than that of BNNR’s 0.932, DisDrugPred’s 0.890, DRRS’s 0.929, SCMFDD’s 0.712, and MBiRW’s 0.911. DDA-SKF also has an AUPR of 0.533, which is only slightly lower than that of BNNR. Therefore, we believe that DDA-SKF has a better, or at least comparable, performance to all state-of-the art methods in novel association prediction. The integration of disease and drug similarity kernels is effective.

**TABLE 1:** Performance comparison analysis using both the novel association test and novel drug test.

Method^a^	AUROC-A^b^	AUPR-A	AUROC-D^c^	AUPR-D
BNNR^d^	0.932	0.589	0.776	0.136
DisDrugPred^e^	0.890	0.070	0.835	0.243
DRRS^e^	0.929	0.140	0.765	0.114
SCMFDD^e^	0.712	0.004	0.733	0.048
MbiRW^e^	0.911	0.129	0.798	0.156
DRIMC^e^	0.956	0.299	0.873	0.278
DDA-SKF	0.937 ± 0.0003^f^	0.533 ± 0.0039	0.845 ± 0.0007	0.270 ± 0.0013

a AUROC: area under receiver operating characteristics; AUPR: area under precision-recall.

b The suffix “-A” indicates that the AUROC and AUPR are obtained using the novel association prediction.

c The suffix “-D” indicates that the AUROC and AUPR are obtained using the novel drug prediction.

d The performance values are taken from Yang et al. (2019a).

e The performance values are taken from Zhang et al. (2020).

f The value is represented as “average ± standard deviation” form for 5 times of repeated cross-validations.

For the novel drug prediction, we evaluated the performance of all models in the benchmarking dataset. The results are recorded in Table 1. DDA-SKF achieved an AUROC of 0.845, which is 8.892, 1.198, 10.458, 15.280, and 5.890%, respectively, higher than that of BNNR’s 0.776, DisDrugPred’s 0.835, DRRS’s 0.765, SCMFDD’s 0.733, and MBiRW’s 0.798. DDA-SKF also obtained an AUPR of 0.270, which is only slightly lower than that of DRIMC. Therefore, we conclude that DDA-SKF outperformed most state-of-the-art methods in novel drug predictions.

In the comparison with PREDICT, LRSSL, and NTSIM methods, 5-fold cross-validation was applied rather than 10-fold cross-validations, as all three existing methods reported their performances in 5-fold cross validations. According to the results in Table 2, DDA-SKF achieved better performances in almost every comparison. DDA-SKF achieved an AUROC of 0.929 and 0.931 for the PREDICT dataset and LRSSL dataset, which are 0.869 and 3.215% higher than that of the second model NTSIM, respectively. As for AUPR, DDA-SKF achieves an AUPR of 0.497 and 0.382 on the PREDICT dataset and LRSSL dataset, which are the best values among the four. For the F1-score, DDA-SKF achieves 0.504 and 0.427 for the PREDICT dataset and LRSSL dataset, which are 25.373 and 26.331% higher than that of NTSIM, respectively. In summary, DDA-SKF achieved better performances in this comparison.

**TABLE 2:** Performance comparison analysis with PREDICT, NTSIM, and LRSSL.

Method	Dataset	AUROC^a^	AUPR^b^	SEN^c^	SPE^d^	PRE^e^	ACC^f^	F1^g^	MCC^h^
PREDICT^i^	PREDICT	0.902	0.151	0.341	0.993	0.091	0.992	0.144	0.163
NTSIM^i^	PREDICT	0.921	0.338	0.368	0.999	0.462	0.998	0.402	0.407
DDA-SKF	PREDICT	0.929 ± 0.0015^j^	0.497 ± 0.0043	0.455 ± 0.0072	0.996 ± 0.0002	0.565 ± 0.0120	0.991 ± 0.0002	0.504 ± 0.0051	0.503 ± 0.0054
LRSSL^i^	LRSSL	0.825	0.179	0.217	0.999	0.199	0.998	0.202	0.203
NTSIM^i^	LRSSL	0.902	0.269	0.308	0.999	0.376	0.999	0.338	0.337
DDA-SKF	LRSSL	0.931 ± 0.0011	0.382 ± 0.0051	0.395 ± 0.0084	0.997 ± 0.0002	0.467 ± 0.0138	0.994 ± 0.0002	0.427 ± 0.0037	0.426 ± 0.0041

a AUROC: area under receiver operating characteristics.

b AUPR: area under precision-recall.

c SEN: sensitivity.

d SPE: specificity.

e PRE: precision.

f ACC: accuracy.

g F1: F1-score.

h MCC: Matthew’s correlation coefficient.

i The performance values are taken from Zhang et al.( 2018a).

j The value is represented as “average ± standard deviation” form for 5 times of repeated cross-validations.

### 3.2 Case Study

Although the prediction performances of DDA-SKF in terms of AUROC and AUPR are slightly lower than those of the best method for the novel association prediction and novel drug prediction, our method can work without enough disease similarities. This is useful when the orphan drugs, whose indication is still very limited, are considered.

In this section, the capability of our model in predicting novel drug–disease associations is tested. We performed three tests based on the PREDICT dataset in this part: one is drug-repositioning prediction, the second is orphan drug indications prediction, and the last is complex disease prediction.

To predict novel indications for all drugs, all known drug–disease associations in the benchmarking dataset were used as the training set, and the unknown drug–disease associations were regarded as the candidate set. DDA-SKF was applied to obtain scores for all candidate drug–disease associations. All candidate associations were ranked according to the prediction scores. Top-ranked candidate associations were identified as novel drug–disease associations.

We verified newly predicted associations by comparing them against the Comparative Toxicogenomics Database (CTD) (Davis et al., 2019). CTD contains curated and inferred chemical–disease relationships, which are divided into marker/mechanism and therapeutic. We only compared the therapeutic relationships for verification. For each of the 593 drugs, we collected the top-5 and the top-20 prediction results. The predictions for all drugs were listed in Supplementary Table S1 and Supplementary Table S2 as OMIM IDs in supplementary materials. In the DDA-SKF prediction results, 156 of top-5 and 377 of top-20 predictions have been confirmed by CTD.

We listed several top-5 prediction results in Table 3. Although some top-ranked predictions had not been verified, we believe that these may provide new indications for approved drugs. For example, vincristine for B chronic lymphocytic leukemia (CLL), cisplatin for acute lymphoblastic leukemia (ALL), and methotrexate for neuroblastoma. These articles provide some possible hints that consist of our predictions (Lj et al., 1997; Vilpo et al., 2000; Lau et al., 2015).

**TABLE 3:** Top five candidate diseases for typical drugs.

Drug	Disease	OMIM ID	Evidence
Vincristine	B-cell chronic lymphocytic leukemia	151400	NA^a^
Vincristine	Small-cell carcinoma	182280	CTD^b^
Vincristine	Mycosis fungoides	254400	CTD
Vincristine	Testicular neoplasms	273300	CTD
Vincristine	Urinary bladder neoplasms	109800	CTD
Cisplatin	Alveolar rhabdomyosarcoma	268220	NA
Cisplatin	Wilms’ tumor	194070	CTD
Cisplatin	Stomach neoplasms	137215	CTD
Cisplatin	Acute lymphoblastic leukemia	247640	NA
Cisplatin	Colorectal neoplasms	114500	CTD
Fluorouracil	Esophageal neoplasms	133239	CTD
Fluorouracil	Renal cell carcinoma	144700	CTD
Fluorouracil	Acute lymphoblastic leukemia	247640	NA
Fluorouracil	Prostatic neoplasms	176807	CTD
Fluorouracil	Acute myelocytic leukemia	246470	NA
Methotrexate	B-cell chronic lymphocytic leukemia	151400	CTD
Methotrexate	Neuroblastoma	256700	NA
Methotrexate	Wilms’ tumor	194070	NA
Methotrexate	Lung neoplasms	211980	CTD
Methotrexate	Glioma	137800	CTD
Paclitaxel	Mismatch repair cancer syndrome 1	276300	NA
Paclitaxel	Prostatic neoplasms	176807	CTD
Paclitaxel	Testicular germ cell tumor	273300	CTD
Paclitaxel	Stomach neoplasms	137215	CTD
Paclitaxel	Cutaneous malignant melanoma, 1	155600	NA

a NA: not available on the Comparative Toxicogenomics Database.

b CTD: Available on the Comparative Toxicogenomics Database.

In particular, DDA-SKF can perform drug–disease association prediction without disease similarities. This may be important in studying some orphan drugs. Since the indications of orphan drugs are usually very limited, the similarities between diseases may be misleading. Therefore, by excluding the disease similarities from the model, DDA-SKF should have more potential in finding new indications for orphan drugs, which may eventually decrease the cost of orphan drug utilization.

We selected several orphan drugs from Orphanet (http://www.orpha.net), including celecoxib, methotrexate, and doxorubicin. For each orphan drug, all known drug–disease associations were removed. The orphan drug would have no association information during the process of prediction. We fed the drug similarity kernels with orphan drug information and the aforementioned association matrix to our model. The results of top-20 predictions for each orphan drug were recorded in Supplementary Table S3 in supplementary materials. The top-5 predicted results are summarized in Table 4. These successful prediction instances further confirm that DDA-SKF has the potential to predict novel indications for orphan drugs without disease similarity information.

**TABLE 4:** Top five candidate diseases for typical orphan drugs.

Orphan drug	Disease	OMIM ID	Evidence
Celecoxib	Osteoarthritis susceptibility 2	140600	CTD^a^
Celecoxib	Osteoarthritis susceptibility 1	165720	CTD
Celecoxib	Progressive pseudorheumatoid dysplasia	208230	NA^b^
Celecoxib	Mitochondrial recessive ataxia syndrome	607459	NA
Celecoxib	Osteoarthritis susceptibility 3	607850	CTD
Methotrexate	Mismatch repair cancer syndrome 1	276300	NA
Methotrexate	Breast neoplasms	114480	CTD
Methotrexate	Acute lymphoblastic leukemia	247640	NA
Methotrexate	Autoimmune diseases	109100	CTD
Methotrexate	Pyogenic arthritis–pyoderma gangrenosum–acne	604416	NA
Doxorubicin	Mismatch repair cancer syndrome 1	276300	NA
Doxorubicin	Acute lymphoblastic leukemia	247640	NA
Doxorubicin	Dohle bodies and acute leukemia	223350	NA
Doxorubicin	Breast neoplasms	114480	CTD
Doxorubicin	Acute myeloid leukemia	601626	CTD

a CTD: available on the Comparative Toxicogenomics Database.

b NA: not available on the Comparative Toxicogenomics Database.

Last, several commonly studied complex diseases were chosen to evaluate DDA-SKF. For instance, Alzheimer’s disease (AD), Parkinson’s disease (PD), and amyotrophic lateral sclerosis (ALS) were considered. We listed the top-5 predicted results in Table 5 along with the literature evidences. For each disease, the uncovered associations will be ranked based on the prediction scores, and the top-ranked unknown associations were identified as the prediction results. The AD and PD results were obtained on the PREDICT dataset, while the ALS results were obtained using the LRSSL dataset. These results indicate that DDA-SKF has the potential to uncover new drugs for these complex diseases.

**TABLE 5:** Top five candidate drugs for complex diseases.

Disease	Drug	Evidence^a^
Alzheimer’s disease	Pyridostigmine	Agnoli et al. (1983)
Alzheimer’s disease	Benzatropine	NA^b^
Alzheimer’s disease	Scopolamine	Chen and Yeong, (2020)
Alzheimer’s disease	Carbidopa	El-Moursy et al. (2009)
Alzheimer’s disease	Pramipexole	Bennett et al. (2016)
Parkinson’s disease	Biperiden	Kostelnik et al. (2017)
Parkinson’s disease	Levodopa	LeWitt, (2015)
Parkinson’s disease	Bromocriptine	Lieberman and Goldstein, (1985)
Parkinson’s disease	Trihexyphenidyl	Jilani et al. (2021)
Parkinson’s disease	Rivastigmine	Espay et al. (2021)
Amyotrophic lateral sclerosis	Baclofen	Boussicault et al. (2020)
Amyotrophic lateral sclerosis	Mexiletine	Adiao et al. (2020)
Amyotrophic lateral sclerosis	Colchicine	Mandrioli et al. (2019)
Amyotrophic lateral sclerosis	Ranolazine	Djamgoz and Onkal, (2013)
Amyotrophic lateral sclerosis	Prilocaine	NA

a Evidence: Evidences from the literature.

b NA: Evidences are not available.

### 3.3 Comparison With Single Similarity

**FIGURE 2:**
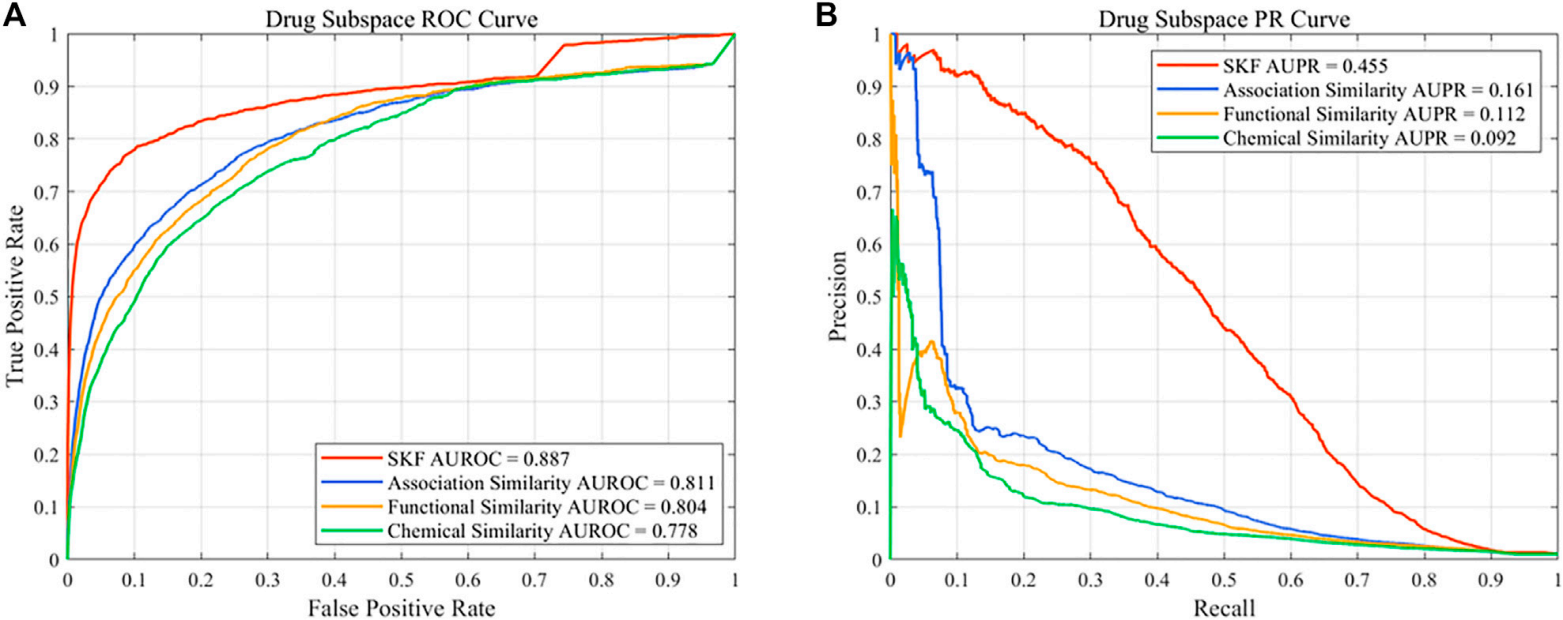
Performance of the single similarity and SKF in the drug subspace of the PREDICT dataset. **(A)** ROC curve and **(B)** PR curve.

**FIGURE 3:**
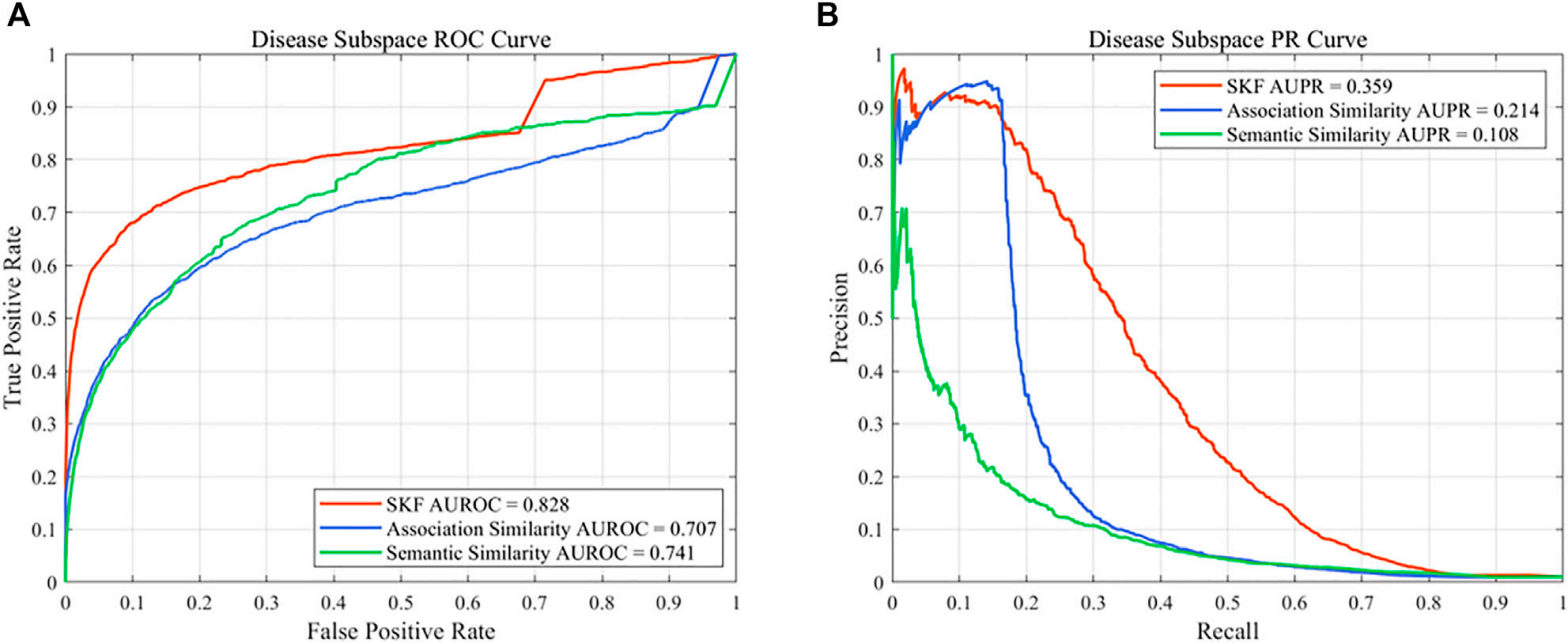
Performance of the single similarity and SKF in the disease subspace of the PREDICT dataset. **(A)** ROC curve and **(B)** PR curve.

In this work, different drug similarity kernels were integrated as a drug comprehensive similarity kernel in the drug subspace. Also, different disease similarity kernels were integrated as a disease comprehensive similarity kernel. To demonstrate the effectiveness in integrating multiple similarity kernels, we calculated the values of AUROC and AUPR with every single similarity kernel on the PREDICT dataset. The results are shown in Figure 2 and Figure 3. We also compared the effectiveness of the multiple similarity kernels fusion and all single similarity kernels. The results are recorded in Table 6. We can see that the comprehensive kernel produced higher performances than every single kernel.

**TABLE 6:** Performance comparison between the multiple similarity fusion and every single similarity.

Space	Similarity	AUROC^a^	AUPR^b^	SEN^c^	SPE^d^	PRE^e^	ACC^f^	F1^g^	MCC^h^
Drug	Chemical	0.778 ± 0.0017^i^	0.092 ± 0.0001	0.158 ± 0.0178	0.991 ± 0.0021	0.164 ± 0.0256	0.983 ± 0.0019	0.159 ± 0.0010	0.151 ± 0.0029
Drug	Functional	0.804 ± 0.0010	0.112 ± 0.0024	0.221 ± 0.0078	0.989 ± 0.0008	0.173 ± 0.0049	0.981 ± 0.0007	0.194 ± 0.0010	0.186 ± 0.0011
Drug	Association	0.811 ± 0.0012	0.161 ± 0.0037	0.261 ± 0.0145	0.989 ± 0.0010	0.200 ± 0.0063	0.981 ± 0.0009	0.226 ± 0.0024	0.219 ± 0.0032
Drug	SKF	0.887 ± 0.0018	0.455 ± 0.0035	0.458 ± 0.0115	0.995 ± 0.0003	0.515 ± 0.0112	0.990 ± 0.0001	0.485 ± 0.0040	0.481 ± 0.0038
Disease	Semantic	0.741 ± 0.0015	0.108 ± 0.0021	0.185 ± 0.0205	0.991 ± 0.0026	0.188 ± 0.0297	0.983 ± 0.0023	0.184 ± 0.0044	0.177 ± 0.0059
Disease	Association	0.707 ± 0.0026	0.214 ± 0.0057	0.168 ± 0.0049	0.999 ± 0.0001	0.846 ± 0.0388	0.991 ± 0.0001	0.280 ± 0.0074	0.374 ± 0.0109
Disease	SKF	0.828 ± 0.0020	0.359 ± 0.0033	0.345 ± 0.0073	0.996 ± 0.0003	0.503 ± 0.0145	0.990 ± 0.0002	0.409 ± 0.0020	0.411 ± 0.0027

a AUROC: area under receiver operating characteristics.

b AUPR: area under precision-recall.

c SEN: sensitivity.

d SPE: specificity.

e PRE: precision.

f ACC: accuracy.

g F1: F1-score.

h MCC: Matthew’s correlation coefficient.

i The value is represented as “average ± standard deviation” form for 5 times of repeated cross-validations.

## 4 Conclusion

In this study, we proposed DDA-SKF (drug–disease associations prediction based on the similarity kernels fusion) for predicting drug–disease associations. Several similarity kernels of drugs and diseases were integrated. The SKF method has a better, or at least comparable, performance than the existing methods, in terms of AUROC and AUPR. The novel drug prediction test indicated that DDA-SKF can identify potential indications of approved drugs, which may be useful in drug repositioning. The DDA-SKF can also make predictions without disease similarity. This allows it to be applied on orphan drugs, which is useful in exploring potential indications of such drugs. The evaluation on several complex diseases illustrates that our method can provide valuable information and potential indications for clinical studies. However, it should be noted that there is still a room for further improvement. One is to integrate similarities from more biological knowledge, and the other is to integrate information of the drug-target information. Due to the limited time and resources of this study, these works will be conducted in future.

## Data Availability

Publicly available datasets were analyzed in this study. These data can be found here: https://github.com/GCQ2119216031/DDA-SKF.
